# Successful treatment of primary bone marrow Hodgkin lymphoma with brentuximab vedotin: a case report and review of the literature

**DOI:** 10.1186/s13256-018-1693-0

**Published:** 2018-05-30

**Authors:** Keiki Nagaharu, Masahiro Masuya, Yuki Kageyama, Takanori Yamaguchi, Ryugo Ito, Keiki Kawakami, Masafumi Ito, Naoyuki Katayama

**Affiliations:** 1 Department of Hematology and Oncology, Suzuka General Hospital, Mie, Japan; 2 Department of Hematology and Oncology, Mie University Graduate School of Medicine, Edobashi 2-174, Tsu, Mie 514-8507 Japan; 3 Department of Pathology, Japanese Red Cross Nagoya First Hospital, Aichi, Japan

**Keywords:** Epstein-Barr virus, Primary bone marrow Hodgkin lymphoma

## Abstract

**Background:**

Hodgkin lymphoma usually presents with sequential enlargement of peripheral lymph nodes, and bone marrow invasion rarely occurs (approximately 3–5%). However, several cases have been reported as “primary” bone marrow Hodgkin lymphoma, especially among patients with human immunodeficiency virus and the elderly. This type of Hodgkin lymphoma is characterized by no peripheral lymphadenopathies and has been reported to have poorer prognosis.

**Case presentation:**

A 38-year-old Japanese man was admitted to our hospital because of fever of unknown origin and pancytopenia without lymphadenopathies. Bone marrow examination revealed Hodgkin cells mimicking abnormal cells. These were positive for CD30, EBER-1, CD15, PAX-5, and Bob-1 and negative for Oct-2, CD3, CD20, surface immunoglobulin, CD56. On the basis of systemic evaluation and bone marrow examination, he was diagnosed with primary bone marrow Hodgkin lymphoma. We initiated therapy with DeVIC (dexamethasone, etoposide, ifosfamide, and carboplatin) therapy, but remission was not achieved. Then, the patient was treated with brentuximab vedotin combined with systemic chemotherapy (Adriamycin, vinblastine and dacarbazine), which was effective.

**Conclusions:**

There is no established treatment strategy for Hodgkin lymphoma, and therapeutic outcomes using ABVD (Adriamycin, bleomycin, vinblastine and dacarbazine)-like or CHOP (cyclophosphamide, Adriamycin, vincristine, and prednisone)-like regimens are reportedly poor. Only a few patients have been reported to achieve long-term remission. Through this case report, we suggest an alternative therapeutic option for primary bone marrow Hodgkin lymphoma.

## Background

Hodgkin lymphoma (HL), one of the most common lymphoproliferative diseases, characteristically presents with progressive and sequential enlargement of peripheral lymph nodes [1]. Bone marrow (BM) invasion rarely occurs in patients with HL (approximately 3–5%) and typically only in those with advanced disease [2]. However, Shah *et al.* [3] reported a rare case of a patient with “primary bone marrow” Hodgkin lymphoma (PBMHL) with human immunodeficiency virus (HIV). PBMHL has also been reported in both HIV-positive and HIV-negative patients.

Epstein-Barr virus (EBV) is believed to play a causative role in HIV-associated HL [4]. Use of *in situ* hybridization or immunohistochemical staining has revealed that approximately 40% of patients with non-HIV-associated HL [5] and 75–78% of patients with HIV-associated HL are EBV-positive [4, 6]. The precise mechanism by which EBV contributes to development of HL remains unclear. EBV infection has no influence on the prognosis of children with HL [7]. However, some researchers have reported a higher relapse rate after primary treatment in middle-aged patients with EBV-associated HL than in those with non-EBV-associated HL [8]. In patients with refractory HL, dose-dense systemic chemotherapy, brentuximab vedotin (BV), and autologous hematopoietic stem cell transplant are considered salvage treatments. BV, which is a CD30-directed antibody conjugated with monomethyl auristatin E, is an approved treatment for patients with relapsed or refractory HL [9], and it has safely been combined with systemic chemotherapy [10]. In this report, we present a case of a patient with EBV-associated PBMHL who was successfully treated with BV-containing combination chemotherapy.

## Case presentation

A 38-year-old Japanese man was admitted to our hospital because of progressive fever and thrombocytopenia for more than 1 month. His medical history included Burkitt lymphoma (negative for EBV-encoded small ribonucleic acid (RNA)), and he had been treated with hyper-CVAD (cyclophosphamide, vincristine, doxorubicin, and dexamethasone) 12 years before admission. He had achieved complete remission. He was a daily smoker (18 pack-years) and took no daily medications. He had a family history of hypertension and denied having any malignancies.

The patient’s physical examination findings were normal except for small papules on his upper back. Laboratory tests showed pancytopenia, high C-reactive protein, and a negative result for HIV (Table 1). Computed tomography (CT) revealed moderate pulmonary emphysema with no evidence of infection or inflammation. Positron emission tomography/CT was unavailable for financial reasons. A skin biopsy of his rash revealed no malignant change. BM examination revealed hemophagocytosis without abnormal cells. Plasma viral deoxynucleic acid (DNA) was investigated to identify possible causes of hemophagocytosis, and the patient was found to be EBV DNA-positive (170,000 copies/ml).

**Table 1:** Laboratory findings on admission

Complete blood count		Coagulation		Biochemistry			
WBC	3300/μl	APTT	32.2 s	TP	6.2 g/dl	IgG	1145 mg/dl
Neu	77.00%	PT	60%	Alb	3.8 g/dl	IgA	194 mg/dl
Lym	12.00%	FDP	8.4 μg/μl	AST	18 IU/L	IgM	28 mg/dl
Mo	5.00%	Fibrinogen	442 mg/dl	ALT	17 IU/L	sIL-2 receptor	7020 U/ml
Eos	0.00%			LDH	273 IU/L	β_2_-MG	1.4 μg/dl
Bas	0.00%			γ-GTP	28 IU/L	IL-6	20.6 pg/ml
Aty lym	5.00%			T-Bil	1.3 mg/dl	IFN-γ	6.3 IU/ml
RBC	331 × 10^4^/μl			BUN	13.7 mg/dl	TNF-α	Negative
Hb	10.6 g/dl			Cre	0.68 mg/dl	Procalcitonin	0.06 ng/ml
Hct	31.00%			Na^+^	135 mEq/L	HIV antibody	Negative
MCV	93.7 fl			K^+^	4.4 mEq/L	Parvovirus IgM	Negative
Plt	10.6 × 10^4^/μl			Cl^−^	102 mEq/L	Parvovirus IgG	Positive
CD4^+^ T cells	220/μl			CRP	5.87 mg/dl	HHV-6 IgM	Negative
				Fe	46 mg/dl	HHV-6 IgG	Positive
				Ferritin	771 ng/ml	C7-HRP	Negative
						EB EA IgG	Negative
							(EIA index = 0.4)
						EB VCA IgM	Negative
							(EIA index = 0.5)
						EB VCA IgG	Positive
							(EIA index = 12.1)
						EBNA IgG	Positive
							(EIA index = 2.5)
						EBV DNA	4200 copies/ml

*Abbreviations: WBC* White blood cells, *APTT* Activated partial thromboplastin time, *TP* Total protein, *IgG* Immunoglobulin G, *Neu* Neutrophils, *PT* Prothrombin time, *Alb* Albumin, *IgA* Immunoglobulin A, *Lym* Lymphocytes, *FDP* Fibrinogen degradation product, *AST* Aspartate aminotransferase, *IgM* Immunoglobulin M, *Mo* Monocytes, *ALT* Alanine aminotransferase, *sIL-2* Soluble interleukin 2, *Eos* Eosinophils, *LDH* Lactate dehydrogenase, *β*_*2*_*-MG* β_2_-microglobulin, *Bas* Basophils, *γ-GTP* γ-Glutamyltransferase, *IL-6* Interleukin 6, *Aty lym* Atypical lymphocytes, *T-Bil* Total bilirubin, *IFN-γ* Interferon-γ, *RBC* Red blood cells, *BUN* Blood urea nitrogen, *TNF-α* Tumor necrosis factor-α, *Hb* Hemoglobin, *Cre* Creatinine, *Hct* Hematocrit, *HIV* Human immunodeficiency virus, *MCV* Mean corpuscular volume, *Plt* Platelets, *CRP* C-reactive protein, *HHV-6* Human herpesvirus 6, *C7-HRP* Cytomegalovirus antigenemia, *EB EA IgG* Epstein-Barr virus early antigen immunoglobulin G, *EB VCA* Epstein-Barr virus viral capsid antigen immunoglobulin M, *EIA* Enzyme immunoassay, *EBNA* Epstein-Barr nuclear antigen

**Fig. 1:**
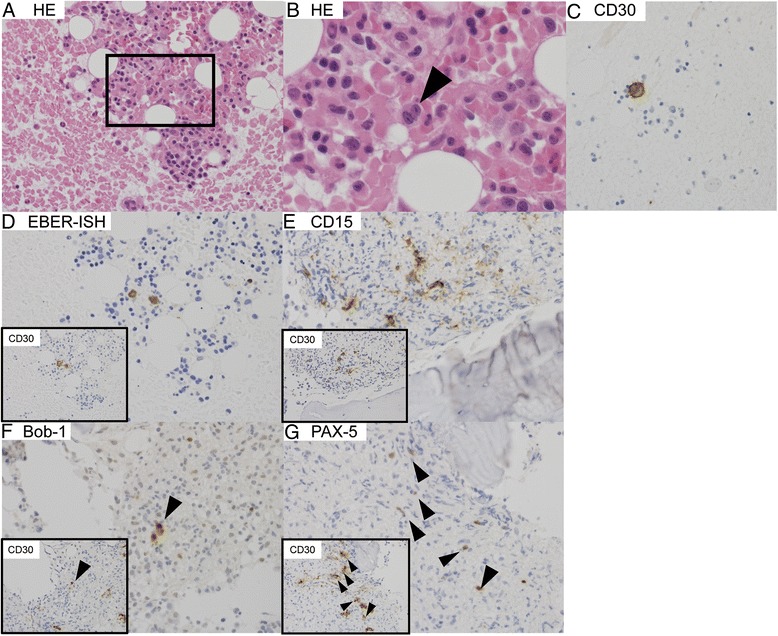
Histopathological findings in bone marrow on presentation. Representative photomicrographs of hematoxylin and eosin (HE) and immunohistochemically stained sections of bone marrow. Rectangular area in (**a**) (HE, × 40) is shown at higher magnification in (**b**) (HE, original magnification × 400). Reed-Sternberg cell-like cells are present (*arrowhead*). These cells are positive for CD30 (**c**, original magnification × 400), Epstein-Barr encoding region *in situ* hybridization (EBER-ISH) (**d**, original magnification × 400), CD15 (**e**, original magnification × 400), Bob-1 (**f**, original magnification × 400), and PAX-5 (**g**, original magnification × 400). The small rectangulars in **d**–**g** show expression of CD30 in the same field. *Arrowheads* in **f** and **g** identify the CD30 positive malignant cells

As recommended by a previous report, the patient’s EBV-associated hemophagocytic lymphohistiocytosis (HLH) was treated with chemotherapy comprising etoposide and dexamethasone [11]. Shortly after initiation of chemotherapy, his white blood cell count recovered to within the normal range, and his plasma EBV DNA became undetectable. However, his EBV DNA turned positive (290 copies/ml), and his white blood cell count declined again 7–9 weeks later. A second BM sample showed infiltration with Reed-Sternberg (RS)-like cells (Fig. 1a, b) that were positive for CD30 (Fig. 1c), EBER-1 (Epstein-Barr encoding region 1) (Fig. 1d), CD15 (Fig. 1e), Bob-1 (Fig. 1d), and PAX-5 (Fig. 1e) but negative for Oct-2, CD3, CD20, surface immunoglobulin, and CD56. Another CT examination showed neither lymphadenopathy nor hepatosplenomegaly. The patient was eventually diagnosed with PBMHL (Ann Arbor stage IVB, International Prognostic Index high-intermediate risk). Previous reports have indicated that PBMHL progresses rapidly and that combination chemotherapy (Adriamycin, bleomycin, vinblastine and dacarbazine [ABVD]-like or cyclophosphamide, Adriamycin, vincristine and prednisone [CHOP]-like regimens) is ineffective, as shown in Table 2. Because some patients with EBV-associated lymphoproliferative disease express P-glycoprotein, which is a multidrug resistance 1 (MDR1) gene product [12], the poor prognosis of PBMHL may be related to the presence of the *MDR1* gene. DeVIC (dexamethasone, etoposide, ifosfamide, and carboplatin) includes ifosfamide and carboplatin, which are MDR-unrelated anticancer agents.

**Table 2:** Compilation of published reports of patients with primary bone marrow Hodgkin lymphoma

Patient background	Age (years)	Sex	CD4 count	CD15	CD30	EB virus	Therapies	Survival (months)	Reference
Non-HIV	20	F	No data	ND	ND	ND	MOPP	9	[15]
Non-HIV	72	F	No data	+	+	EBER1^+^	THP-COP	1	[16]
Non-HIV	64	M	No data^a^	+	+	ND	ABVD	1	[17]
Non-HIV	50	M	No data^b^	+	+	LMP1^+^	ABVD	1	[18]
Non-HIV	66	F	No data^a^	+	+	EBER1^+^	A(B)VD	15	[19]
Non-HIV	68	M	No data^a^	ND	+	EBER1^+^	ND	1	[20]
Non-HIV	89	F	No data^b^	–	+	Negative	ND	ND	[21]
AIDS	58	M	20	+	+	ND	ABVD	2	[22]
AIDS	36	M	31	+	+	ND	ABVD	4	
AIDS	31	M	549	+	+	ND	ABVD	18	
AIDS	49	M	54	+	+	ND	ABVD	114	
AIDS	33	M	104	+	+	ND	EBV^a^	4	
AIDS	34	M	86	+	+	ND	ABVD	3	
AIDS	29	M	193	ND	ND	ND	MOPP	31	[23]
AIDS	55	M	14	ND	ND	ND	ND	ND	[24]
AIDS	43	M	179	+	+	ND	ABVD	ND	[3]
AIDS	26	M	No data	+	+	ND	ND	ND	[25]
AIDS	29	F	No data	+	–	ND	ND	ND	
AIDS	27	M	195	+	+	ND	ND	ND	
AIDS	34	M	17	+	+	Negative	ND	ND	
AIDS	26	M	39	+	+	ND	ND	ND	
AIDS	31	M	123	+	+	ND	ND	ND	
AIDS	49	M	52	+	+	ND	ND	ND	
AIDS	40	M	56	+	+	ND	ND	ND	
AIDS	35	M	104	+	+	LMP1^+^	ND	ND	
AIDS	41	M	237	+	+	ND	ND	ND	
AIDS	51	M	282	+	+	LMP1^+^	ND	ND	
AIDS	42	M	198	+	+	ND	ND	ND	

*Abbreviations: AIDS* Acquired immunodeficiency syndrome, *HIV* Human immunodeficiency virus, *MOPP* Mechlorethamine, vincristine, procarbazine, and prednisone, *THP-COP* Pirarubicin, cyclophosphamide, vincristine, and prednisolone, *ABVD* Adriamycin, bleomycin, vinblastine and dacarbazine, *ND* No data, *EBER1* Epstein-Barr virus-encoded RNA 1, *LMP1* Latent membrane protein 1, *EB* Epstein-Barr virus

^a^Leukocytopenia (under 3500/μl) but no CD4 count available

^b^Lymphocytopenia (under 1000/μl) but no CD4 count available

In addition, because of coexisting pulmonary emphysema, DeVIC therapy was initiated at 12 weeks from onset. Although DeVIC therapy induced transient recovery of pancytopenia, it recurred with high plasma EBV DNA titers (2300 copies/ml) after the second course of DeVIC therapy (19–20 weeks from onset). Because a third BM examination revealed residual RS cells, the patient’s disease was considered refractory to DeVIC therapy. After giving informed consent, he was further treated with BV (1.2 mg/kg) and AVD (Adriamycin, vinblastine, and dacarbazine) as described in a previous report [12]. His peripheral blood cell count recovered without support of medication, and the RS cells disappeared. After four courses of combined chemotherapy, BV monotherapy was continued for 8 months, during which both soluble interleukin (IL)-2 receptor and plasma EBV DNA titers remained within the normal range. The patient declined autologous hematopoietic stem cell transplant. To date, no evidence of relapse has been detected.

## Discussion

The overall incidence of BM involvement in HL is reportedly 5% [2]. However, approximately 40–50% of patients with HIV-associated HL have BM invasion, and these patients commonly present with an advanced stage of disease (mainly stage IV). PBMHL is characterized by solitary BM invasion with HL. PBMHL has been reported in patients with and without HIV infection (Table 2). Our review of published reports revealed that patients with acquired immunodeficiency syndrome or older patients are more likely to develop PBMHL. Although our patient was HIV-negative, his number of CD4^+^ T cells was low (200/μl). As shown in Table 2, low CD4^+^ T-cell counts (or lymphocytopenia) are common in patients with PBMHL, especially in those with HIV-associated PBMHL, in whom CD4^+^ T-cell counts are reportedly 133 ± 130/μl (range 14–549/μl, median 103/μl).

Our patient developed HLH as the first symptom of HL. Although HL-associated HLH is rare, a previous retrospective study showed that EBV was frequently detected in patients with HL-associated HLH [13]. Patients with HIV-associated HL, in whom HLH is more common, also exhibit a high prevalence of EBV. These findings suggest that patients with HL-associated HLH might have an unclear underlying immune disturbance for EBV. Our patient’s case indicates that clinicians should perform BM biopsies to check for PBMHL in patients with (1) pancytopenia, (2) low CD4^+^ T-cell counts (or lymphocytopenia), and (3) EBV DNA positivity.

As for the prognosis of PBMHL, only a few patients achieve long-term remission (Table 2). When our patient’s PBMHL proved refractory, we selected BV with AVD as salvage therapy for the following reasons. First, because of its rarity, there is no established treatment strategy for this disease, and therapeutic outcomes using ABVD- or CHOP-like regimens are reportedly poor (as shown in Table 2). Second, bleomycin was contraindicated for our patient because of his coexisting moderate emphysema. Third, a combination of BV with AVD was significantly superior to BV with ABVD [10]. Fortunately, our patient completed his planned therapy without relapse. To the best of our knowledge, this is the first reported case of successful treatment of HIV-negative PBMHL with BV.

The association of EBV and HL has been investigated; however, the exact mechanism involved remains unclear. Approximately 40% of patients with non-HIV-associated HL are EBV-positive [5]; the rate of EBV positivity is much higher in patients with HIV-associated HL [4, 6]. In addition to *in situ* hybridization and histopathological staining, plasma EBV DNA titers are also a useful biomarker for monitoring prognosis in patients with EBV-associated HL [14]. In our patient, an increase in EBV DNA titer preceded recurrence of pancytopenia and occurred earlier than the increase in soluble IL-2 receptor titer. Plasma EBV DNA titers may be helpful for early detection of recurrence of PBMHL.

## Conclusions

We present a rare case of a patient with PBMHL without HIV infection. More experience is needed to establish the optimum treatment for this disease. On the basis of our patient’s progress, we propose that combined therapy with BV and AVD could be a therapeutic option for PBMHL.

## Abbreviations

ABVD: Adriamycin, bleomycin, vinblastine and dacarbazine
AIDS: Acquired immunodeficiency syndrome
Alb: Albumin
ALT: Alanine aminotransferase
APTT: Activated partial thromboplastin time
AST: Aspartate aminotransferase
Aty lym: Atypical lymphocytes
AVD: Adriamycin, vinblastine, and dacarbazine
Bas: Basophils
BM: Bone marrow
BUN: Blood urea nitrogen
BV: Brentuximab vedotin
C7-HRP: Cytomegalovirus antigenemia
CHOP: Cyclophosphamide, Adriamycin, vincristine, and prednisone
Cre: Creatinine
CRP: C-reactive protein
CT: Computed tomography
DeVIC: Dexamethasone, etoposide, ifosfamide, and carboplatin
EB: Epstein-Barr virus
EB EA IgG: Epstein-Barr virus early antigen immunoglobulin G
EB VCA: Epstein-Barr virus viral capsid antigen immunoglobulin M
EBER: Epstein-Barr-encoding region
EBER1: Epstein-Barr virus-encoded RNA 1
EBNA: Epstein-Barr nuclear antigen
EBV: Epstein-Barr virus
EIA: Enzyme immunoassay
Eos: Eosinophils
FDP: Fibrinogen degradation product
γ-GTP: γ-Glutamyltransferase
Hb: Hemoglobin
Hct: Hematocrit
HHV-6: Human herpesvirus 6
HIV: Human immunodeficiency virus
HL: Hodgkin lymphoma
HLH: Hemophagocytic lymphohistiocytosis
IFN-γ: Interferon-γ
IgA: Immunoglobulin A
IgG: Immunoglobulin G
IgM: Immunoglobulin M
IL-6: Interleukin 6
LDH: Lactate dehydrogenase
LMP1: Latent membrane protein 1
Lym: Lymphocytes
MCV: Mean corpuscular volume
MDR1: Multidrug resistance 1
β_2_-MG: β_2_-microglobulin
Mo: Monocytes
MOPP: Mechlorethamine, vincristine, procarbazine, and prednisone
ND: No data
Neu: Neutrophils
PBMHL: Primary bone marrow Hodgkin lymphoma
Plt: Platelets
PT: Prothrombin time
RBC: Red blood cells
RS: Reed-Sternberg
sIL-2: Soluble interleukin 2
T-Bil: Total bilirubin
THP-COP: Pirarubicin, cyclophosphamide, vincristine, and prednisolone
TNF-α: Tumor necrosis factor-α
TP: Thymidine phosphorylase
WBC: White blood cells
